# The Appetite−Suppressant and GLP-1-Stimulating Effects of Whey Proteins in Obese Subjects are Associated with Increased Circulating Levels of Specific Amino Acids

**DOI:** 10.3390/nu12030775

**Published:** 2020-03-15

**Authors:** Antonello E. Rigamonti, Roberto Leoncini, Alessandra De Col, Sofia Tamini, Sabrina Cicolini, Laura Abbruzzese, Silvano G. Cella, Alessandro Sartorio

**Affiliations:** 1 Department of Clinical Sciences and Community Health, University of Milan, 20129 Milan, Italy; silvano.cella@unimi.it; 2 Department of Medical Biotechnologies, University of Siena, 53100 Siena, Italy; roberto.leoncini@unisi.it; 3 Experimental Laboratory for Auxo-endocrinological Research, Istituto Auxologico Italiano, IRCCS, 28824 Piancavallo (VB), Italy; a.decol@auxologico.it (A.D.C.); sofia.tamini@gmail.com (S.T.); s.cicolini@auxologico.it (S.C.); sartorio@auxologico.it (A.S.); 4 Division of Auxology and Metabolic Diseases, Istituto Auxologico Italiano, IRCCS, 28824 Piancavallo (VB), Italy; l.abbruzzese@auxologico.it

**Keywords:** whey proteins, amino acids, maltodextrins, satiety, hunger, obesity, appetite, anorexigenic gastrointestinal peptides, GLP-1, PYY

## Abstract

The satiating effect of whey proteins depends upon their unique amino acid composition because there is no difference when comparing whey proteins or a mix of amino acids mimicking the amino acid composition of whey proteins. The specific amino acids underlying the satiating effect of whey proteins have not been investigated to date. Aims and Methods. The aim of the present study was to evaluate the appetite-suppressant effect of an isocaloric drink containing whey proteins or maltodextrins on appetite (satiety/hunger measured by a visual analogue scale or VAS), anorexigenic gastrointestinal peptides (circulating levels of glucagon-like peptide 1 (GLP-1) and peptide tyrosine tyrosine (PYY)) and amino acids (circulating levels of single, total [TAA] and branched-chain amino acids [BCAA]) in a cohort of obese female subjects (*n* = 8; age: 18.4 ± 3.1 years; body mass index, BMI: 39.2 ± 4.6 kg/m^2^). Results. Each drink significantly increased satiety and decreased hunger, the effects being more evident with whey proteins than maltodextrins. Similarly, circulating levels of GLP-1, PYY and amino acids (TAA, BCAA and alanine, arginine, asparagine, citrulline, glutamine, hydroxyproline, isoleucine, histidine, leucine, lysine, methionine, ornithine, phenylalanine, proline, serine, threonine, tyrosine, and valine) were significantly higher with whey proteins than maltodextrins. In subjects administered whey proteins (but not maltodextrins), isoleucine, leucine, lysine, methionine, phenylalanine, proline, tyrosine, and valine were significantly correlated with hunger (negatively), satiety, and GLP-1 (positively). Conclusions. Eight specific amino acids (isoleucine, leucine, lysine, methionine, phenylalanine, proline, tyrosine, and valine) were implicated in the appetite-suppressant and GLP-1-stimulating effects of whey proteins, which may be mediated by their binding with nutrient-sensing receptors expressed by L cells within the gastrointestinal wall. The long-term satiating effect of whey proteins and the effectiveness of a supplementation with these amino acids (i.e., as a nutraceutical intervention) administered during body weight reduction programs need to be further investigated.

## 1. Introduction

The pandemic prevalence of obesity urgently requires effective body weight reduction programs, including appropriate dietetic regimes [1]. In particular, diets with high protein content have been demonstrated to promote weight loss and, parallelly, to impede weight gain [2].

Proteins represent the most satiating macronutrient [3]. Among different sources of proteins, whey proteins seem to be the most effective [4]. Several mechanisms have been invoked to explain the appetite-suppressant and weight-losing effects that whey proteins produce when administered to normal weight or obese subjects [5]: secretion of gastrointestinal anorexigenic peptides [6,7,8,9], stimulation of energy expenditure via an increased thermogenesis [10], and direct actions on specific hypothalamic areas involved in the regulation of food intake [11].

In the last decade, pharmacological research has identified a heterogeneous group of receptors, the so-called nutrient-sensing receptors, capable of recognizing specific nutrients such as amino acids [12]. G-protein-coupled receptors, including T1Rs, GPRC6A, and CaSR are the major amino acid sensing receptors [13,14]. Some of these receptors are expressed by enteroendocrine cells located within the gastrointestinal wall and secrete a plethora of peptides regulating gastrointestinal physiology, including glucometabolic homeostasis and gut-brain axis [15,16]; One example is represented by L cells, predominantly located in the distal part of the gastrointestinal tract, which secrete two potent anorexigenic gastrointestinal peptides, i.e., the glucagon-like peptide 1 (GLP-1) and the peptide tyrosine tyrosine (PYY) [17]. Mixed meals containing proteins and fats represent the most effective physiological stimulus to increase circulating GLP-1 and PYY levels [18].

The appetite-suppressant effect of whey proteins has been associated with parallel increases in circulating levels of both GLP-1 and PYY, and increases in a series of amino acids deriving from the digestion and absorption of α-lactalbumin and β-lactoglobulin, which represent almost 70% of the protein concentration in whey and are digested very quickly after ingestion when compared to other proteins such as casein [7,9,19]. Furthermore, as demonstrated in a study carried out in normal-weight subjects, no difference in appetite-suppressant effects was found when comparing “natural” whey proteins and an “artificial” mix of amino acids mimicking the amino acid composition of whey proteins [20]. This suggests that the appetite-suppressant effect of whey protein may be attributed to the unique amino acid composition of whey proteins, particularly rich in branched-chain amino acids (BCAA) such as isoleucine, leucine, and valine [19].

So far, to the best of our knowledge, no one has identified the specific amino acids that are associated with suppression of appetite and, parallelly, with stimulation of gastrointestinal anorexigenic peptides when whey proteins are administered to obese subjects who present an alteration of the central and peripheral regulation of food intake as compared to the normal-weight counterparts [21,22,23,24,25,26,27,28,29]. Such information may be useful to produce a nutraceutical/pharmacological/biotechnological mix of amino acids to include in the dietetic regimen of a body weight reduction program (BWRP) [2].

Therefore, the aim of the present study was to identify the amino acids that (1) increase in the plasma after intake of a drink containing whey proteins, (2) suppress appetite by decreasing hunger and increasing satiety, and (3) stimulate secretion of GLP-1 and PYY. Our hypothesis is that only a limited number of amino acids deriving from digestion and absorption of whey proteins meet all the three conditions. To validate the results of the present study, obese subjects were recruited; additionally, an isocaloric drink containing maltodextrins was used as comparator of those containing whey proteins.

## 2. Materials and Methods

### 2.1. Patients and Experimental Protocol

Eight obese young women (age: 18.4 ± 3.1 years; body mass index, BMI: 39.2 ± 4.6 kg/m^2^; fat-free mass, FFM: 54.1 ± 5.6%; fat mass, FM: 45.9 ± 5.6%) were recruited among patients hospitalized at the Division of Auxology (aged < 18 yrs) and Metabolic Diseases (aged > 18 yrs), Istituto Auxologico Italiano, Piancavallo (VB), for a multidisciplinary integrated BWRP, entailing energy restricted diet, psychological counselling, physical rehabilitation, and nutritional education. The study was completed in the first 5 days of hospitalization (diagnostic phase) before starting the BWRP period, in order to avoid any carryover effects due to weight loss or changes in diet and physical activity. Subjects having any disease apart from morbid obesity or taking any drugs as part of any other treatment regimen were excluded. Furthermore, subjects were selected as having stable weight that had not varied by more than 5 kg in the previous month; all women in this study were eumenorrheic.

The participants underwent two tests consisting of the oral administration of a drink containing either whey proteins (45 g of Enervit Gymline Muscle 100% whey protein isolate cacao, Enervit spa, Erba, Italy, corresponding to 715 kJ) or maltodextrins (43 g of Enervit Maltodestrine Sport, Enervit spa, Erba, Italy, corresponding to 715 kJ). Each powdered formula was dissolved in 300 mL of semi-skimmed milk; at the end of preparation, each dose of formula corresponded to 585 kJ, for a total of 1300 kJ of metabolizable energy (ME). Testing was done in a randomized order and crossover design, starting from 8.00 AM after 12 h of overnight fasting. Each test was carried out on separate days, with a washout period of at least 2 days.

The drink was consumed in three 100 mL doses administered within 15 min (100 mL every 5 min over 15 min). Each drink was prepared with the same color and taste (by using cacao powder) in order to avoid possible visual and taste conditioning. Blood samples were drawn from all participants at T0 (baseline, before administering the drink), T60 (60 min), and T120 (120 min). By using a visual analogue scale (VAS), appetite (satiety and hunger) was evaluated at the same times before blood sampling (i.e., T0, T60, and T120). Subjects were asked to rate their satiety and hunger on a 10-cm line with labels at the extremities indicating the most negative and the most positive ratings.

The experimental protocol was approved by the local Ethical Committee (research project code: 01C723; acronym: PROLATPEPOB), and all subjects (or their parents) gave their written consent after being fully informed of the nature and procedures of the study.

### 2.2. Blood Sampling and Biochemical Measurements

Blood was collected in tubes with or without anticoagulant (EDTA). Plasma or serum was separated by centrifugation and stored at −20 °C.

Total plasma PYY level, including both PYY_1−36_ and PYY_3−36_, was measured by a commercially available ELISA kit for PYY (Millipore, Saint Charles, MO, USA). The sensitivity of the method was 6.5 pg/mL; intra- and inter-assay coefficients of variation (CVs) were 2.66% and 6.93%, respectively.

Total plasma GLP-1 level, including GLP-1_7−36 amide_, GLP-1_7−37_, GLP-1_9−36 amide_, GLP-1_9−37_, GLP-1_1−36 amide_, and GLP-1_1−37_, was measured by ELISA (Millipore, Saint Charles, MO, USA). A DPP-IV (dipeptidyl protease IV) inhibitor (protease inhibitor cocktail, Sigma-Aldrich−Merck, Darmstadt, Germany) was added to tubes (50 µl) to prevent the breakdown of GLP-1. The sensitivity of the method was 1.5 pmol/L; intra- and inter-assay CVs were 1% and <12%, respectively.

Amino acids (alanine, arginine, asparagine, aspartate, citrulline, glutamate, glutamine, glycine, hydroxyproline, isoleucine, histidine, leucine, lysine, methionine, ornithine, phenylalanine, proline, serine, taurine, threonine, tyrosine, and valine) were analyzed by ion exchange chromatography with post column derivatization with Ninhydrin, using Biochrom 30+ Amino Acids Analyzer (Biochrom Ltd., Cambridge, UK).

### 2.3. Statistical Analyses

The Sigma Stat 3.5 statistical software package was used for data analyses. GraphPad Prisma 5.0 software was used for plotting data.

The Shapiro−Wilk test showed that all parameters were normally distributed.

Results are reported as mean ± SD (standard deviation). The responses in PYY, GLP-1, amino acids (single, total and BCAA), and VAS scores for hunger and satiety were evaluated as continuous variables for each experimental group (whey proteins vs. maltodextrins) and times (T60 and T120 vs. T0). Furthermore, area under the curve was calculated from T0 to T120 by using the trapezoid method (AUC_T0−T120_).

All parameters (PYY, GLP-1, amino acids [total and BCAA], and VAS scores for hunger and satiety) were compared within each experimental group (whey proteins or maltodextrins), over sampling times (intra-group analysis), and between the two experimental groups (whey proteins vs. maltodextrins) for any sampling time (inter-group analysis) by using a two-way ANOVA with repeated measures (with the two factors time and group and the interaction time × group), followed by the post hoc Tukey’s test. A one-way ANOVA with repeated measures, followed by the post hoc Tukey’s test, was used to compare the responses of PYY, GLP-1, amino acids [single, total and BCAA], and VAS scores for hunger and satiety.

Pearson’s coefficient was calculated to correlate each single amino acid with PYY, GLP-1, and VAS scores for hunger and satiety (all data within the same experimental group).

A level of significance of *p* < 0.05 was used for all data analyses.

## 3. Results

**Figure 1:**
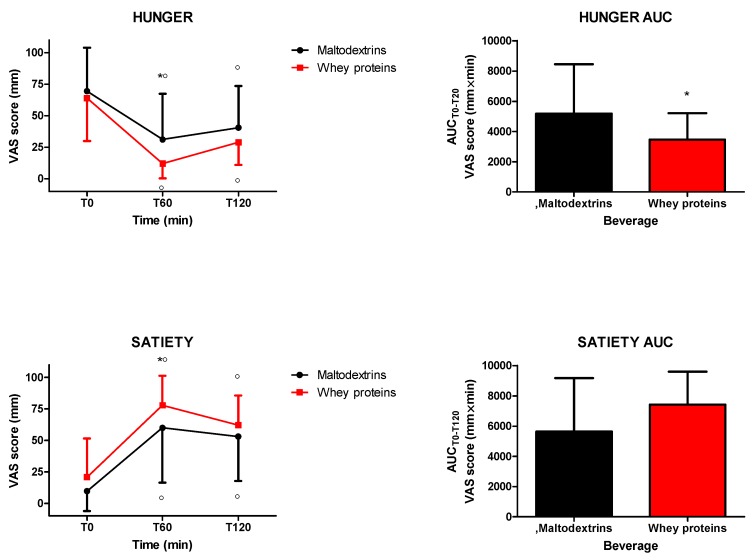
Changes of visual analogue scale (VAS) ratings of hunger (top panel) and satiety (bottom panel) in obese subjects after the intake of a drink (completely within 15 min starting at T0), containing whey proteins or maltodextrins. Values (curves on the left side and areas under the curve from T0 to T120 [AUC_T0−T120_] on the right side) are expressed as mean ± SD. The number of subjects was 8. ○ *p* < 0.05 vs. the corresponding T0 value; * *p* < 0.05 vs. the corresponding value of the maltodextrins-treated group. One- or two-way ANOVA with repeated measures (with the single factor of group, or two factors of time and group and the interaction time × group, respectively), followed by the post hoc Tukey’s test, was used, when appropriate.

The intake of isocaloric drinks containing whey proteins or maltodextrins significantly increased and reduced satiety and hunger, respectively (satiety: 0 min vs. 60 and 120 min for both drinks, *p* < 0.05; hunger: 0 min vs. 60 and 120 min for both drinks, *p* < 0.05). Whey proteins induced more satiety and less hunger (satiety: *p* < 0.05 at 60 min vs. maltodextrins; hunger: *p* < 0.05 at 60 min vs. maltodextrins) (Figure 1). When considering hunger and satiety responses in terms of AUC_T0−T120_, the comparison between the two groups was significantly different only for hunger (*p* < 0.05) (Figure 1).

**Figure 2:**
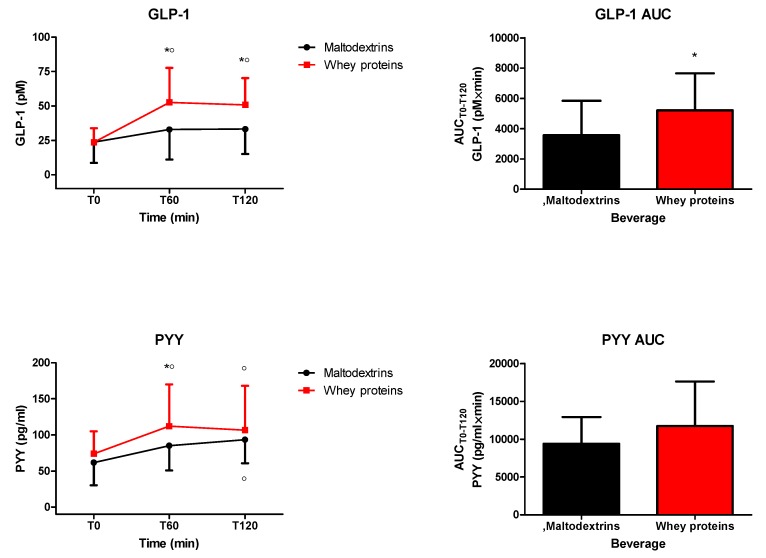
Changes of circulating levels of glucagon-like peptide 1 (GLP-1) (top panel) and peptide tyrosine tyrosine (PYY) (bottom panel) in obese subjects after the intake of a drink (completely within 15 min starting at T0), containing whey proteins or maltodextrins. Values (curves on the left side and areas under the curve from T0 to T120 [AUC_T0−T120_] on the right side) are expressed as mean ± SD. The number of subjects was 8. ○ *p* < 0.05 vs. the corresponding T0 value; * *p* < 0.05 vs. the corresponding value of the maltodextrins-treated group. One- or two-way ANOVA with repeated measures (with the single factor of group, or two factors of time and group, and the interaction time × group, respectively), followed by the post hoc Tukey’s test, was used, when appropriate.

The intake of each drink significantly increased circulating levels of GLP-1 (0 min vs. 60 and 120 min only for the drink containing whey proteins, *p* < 0.05) (Figure 2). Whey proteins induced higher circulating levels of GLP-1 (*p* < 0.05 at 60 and 120 min vs. maltodextrins), an effect confirmed even when considering GLP-1 responses in terms of AUC_T0−T120_ (*p* < 0.05) (Figure 2). Furthermore, the intake of each drink significantly increased circulating levels of PYY (0 min vs. 60 and 120 min for the drink containing whey proteins, and only 120 min for the drink containing maltodextrins, *p* < 0.05) (Figure 2). Whey proteins induced higher circulating levels of PYY (*p* < 0.05 at 60 min vs. maltodextrins) (Figure 2). This significance was missing when considering PYY responses in terms of AUC_T0−T120_ (Figure 2).

**Figure 3:**
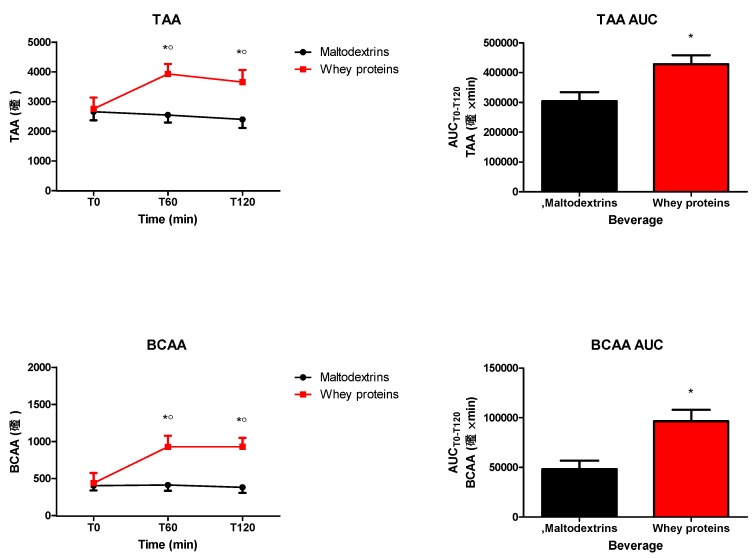
Changes of total amino acids (TAA) (top panel) and branched-chain amino acids (BCAA) (bottom panel) in obese subjects after the intake of a drink (completely within 15 min starting at T0), containing whey proteins or maltodextrins. Values (curves on the left side and areas under the curve from T0 to T120 [AUC_T0−T120_] on the right side) are expressed as mean ± SD. The number of subjects was 8. ○ *p* < 0.05 vs. the corresponding T0 value; * *p* < 0.05 vs. the corresponding value of the maltodextrins-treated group. One- or two-way ANOVA with repeated measures (with the single factor of group or two factors of time and group, and the interaction time × group, respectively), followed by the post hoc Tukey’s test, was used, when appropriate.

The intake of the drink containing whey proteins (but not maltodextrins) significantly increased circulating levels of TAA and BCAA (TAA: 0 min vs. 60 and 120 min, *p* < 0.05; BCAA: 0 min vs. 60 and 120 min, *p* < 0.05) (Figure 3). Whey proteins induced a significantly higher increase of both TAA and BCAA (*p* < 0.05 at 60 and 120 min vs. maltodextrins). These effects were confirmed even when considering TAA and BCA responses in terms of AUC_T0−T120_ (p<0.05) (Figure 3). The AUCs_T0−T120_ of alanine, arginine, asparagine, citrulline, glutamine, hydroxyproline, isoleucine, histidine, leucine, lysine, methionine, phenylalanine, ornithine, proline, serine, threonine, tyrosine, and valine were significantly higher after the intake of the drink containing whey proteins than maltodextrins (*p* < 0.05), being not different the AUCs_T0−T120_ of aspartate, taurine, glutamate and glycine between the two groups (Table 1).

When considering all data from obese subjects administered with the drink containing maltodextrins, significant positive correlations of VAS scores for hunger were found with citrulline and tyrosine (*p* < 0.05) (Table 2). A significant negative correlation of VAS scores for satiety was observed only with citrulline (*p* < 0.05) (Table 2). Significant negative correlations of circulating levels of PYY were found with arginine, hydroxyproline, serine, and threonine (*p* < 0.05) (Table 2). Finally, there were significant positive correlations of circulating levels of GLP-1 with arginine, lysine, methionine, phenylalanine, and proline, but a negative correlation with citrulline (*p* < 0.05) (Table 2). Therefore, after the intake of maltodextrins, citrulline was the unique amino acid having a triple correlation towards an appetite-stimulating effect (i.e., positive with hunger and negative with satiety and GLP-1).

When considering all data from obese subjects administered with the drink containing whey proteins, significant negative correlations of VAS scores for hunger were found with glutamate, isoleucine, leucine, lysine, methionine, phenylalanine, proline, tyrosine, and valine (*p* < 0.05) (Table 2). Significant positive correlations of VAS scores for satiety were observed with arginine, isoleucine, leucine, lysine, methionine, phenylalanine, proline, tyrosine, and valine (*p* < 0.05) (Table 2). A significant positive correlation of circulating levels of PYY was found only with glutamate (*p* < 0.05) (Table 2). Finally, there were significant positive correlations of circulating levels of GLP-1 with asparagine, hydroxyproline, isoleucine, leucine, lysine, methionine, phenylalanine, proline, threonine, tyrosine, and valine (*p* < 0.05) (Table 2). Therefore, after the intake of whey proteins, the unique amino acids having a triple correlation with an appetite-suppressant effect (i.e., negative with hunger and positive with satiety and GLP-1) were isoleucine, leucine, lysine, methionine, phenylalanine, proline, tyrosine, and valine (Table 2).

## 4. Discussion

In the present study, two drinks containing whey proteins or maltodextrins, administered to obese subjects, were compared by measuring VAS scores for hunger and satiety and circulating levels of PYY and GLP-1, two gastrointestinal anorexigenic peptides, and of amino acids (single, TAA and BCAA). Evaluation of the parameters was performed from T0 (before the intake of whey proteins or maltodextrins) until T120 (i.e., at 120 min after).

Whey proteins and maltodextrin decreased hunger and increased satiety, with the appetite-suppressant effect of whey proteins being higher than that of maltodextrins. This finding was congruent with an increase in circulating levels of GLP-1 and PYY, which was higher in the group treated with whey proteins than maltodextrins. The intake of whey proteins (vs. maltodextrins) was followed by a higher increase of all the measured amino acids, with the exclusion of aspartate, glutamate, glycine, and taurine. Importantly, after the intake of whey proteins, circulating levels of a limited number of amino acids were correlated with the appetite-suppression (decrease/increase in hunger/satiety, respectively) and the stimulation of GLP-1 secretion, i.e., isoleucine, leucine, lysine, methionine, phenylalanine, proline, tyrosine, and valine. When considering obese subjects administered with maltodextrins, no amino acid was shown to be endowed with these properties, i.e., appetite-suppression and GLP-1-stimulation. When considering obese subjects administered with whey proteins, there was no correlation between circulating levels of PYY and any amino acid apart from glutamate.

The cellular and molecular mechanisms underlying the appetite-suppressant and GLP-1 stimulating effects of some (and not other) amino acids are only partially known.

In the last decade, pharmacological research has focused on the characterization of specialized receptors, so-called nutrient-sensing receptors, which, expressed predominantly within the gastrointestinal wall, recognize nutrients resulting from digestion of ingested foods [30].

Nutrient-sensing receptors such as T1Rs, GPRC6A, and CaSR, are G-protein-coupled receptors with a different ligand-specificity/affinity for each amino acid [13,14,31]. Furthermore, different amino acids may (promiscuously) bind with the same nutrient-sensing receptor but activate divergent post-receptor pathways [32]. Though there is a limited number of studies investigating cell expression of each nutrient-sensing receptor, L cells (i.e., the neuroendocrine cells that secrete GLP-1) may be endowed with only some of the nutrient-sensing receptors [17].

Therefore, based on the results of the present study, our hypothesis is that isoleucine, leucine, lysine, methionine, phenylalanine, proline, tyrosine, and valine are the unique amino acids derived from digestion and absorption of whey proteins that, at pharmacological levels, stimulate L cells to secrete GLP-1, after binding and activating specific nutrient-sensing receptors. The ensuing increased circulating levels of GLP-1 result in an anorexigenic response, presumably due to direct action of the peptide in hypothalamic areas [33].

Though our hypothesis may be intriguing, our reasoning is biased by some limitations, which should be mentioned here.

First of all, circulating levels of amino acids are a consequence not only of the presence of that amino acid in the unique amino acid composition of whey proteins, but also of the relative concentration of that amino acid among all the amino acids derived from digestion and absorption of whey proteins within the gastrointestinal lumen [7]. Therefore, we cannot rule out that increasing the concentration of any single amino acid (e.g., administering more than 45 g of whey proteins in the drink) results in the acquisition of appetite-suppressant and GLP-1-stimulating effects of that amino acid. This is in accordance with the pharmacological concept of dose/effect and the finding that the satiating effect of whey proteins is dose-dependent [34].

Second, the appetite suppression exerted by some amino acids does not depend exclusively upon stimulation of GLP-1 secretion [5]. In this respect, BCAA (isoleucine, leucine, and valine) are reported to act directly at the hypothalamic level by inhibiting orexigenic pathways and/or activating anorexigenic pathways [35,36,37,38]. Furthermore, other gastrointestinal peptides than the PYY and GLP-1 measured in the present study may be involved in the appetite-suppressant effect of whey proteins, including decrease of circulating levels of orexigenic peptides such as ghrelin [39].

Third, other components of whey proteins may be invoked to explain the appetite-suppressant effect. For example, bioactive peptides have been demonstrated to be released during digestion/hydrolysis of whey proteins, and to possess additional pharmacological properties such as inhibition of dipeptidyl peptidase IV (DPP-IV), which is associated with a prolongation of GLP-1 half-life [40].

Despite the above-mentioned limitations, the results of the present study undoubtedly support our hypothesis that at least a part of the appetite-suppression of whey proteins is mediated by eight specific amino acids (isoleucine, leucine, lysine, methionine, phenylalanine, proline, tyrosine, and valine). Further studies are mandatory to compare appetite-suppression and GLP-1-stimulation between intact whey proteins and the mix of the eight identified amino acids. The choice of the dose of each amino acid should be accurate to obtain the same pharmacokinetic profile after the intake of the two drinks: the one containing whey proteins, and the other the mix of the eight amino acids.

In the present study, PYY appeared to have a negligible role in the appetite-suppressant effect of the eight identified amino acids. In fact, among isoleucine, leucine, lysine, methionine, phenylalanine, proline, tyrosine, and valine, no amino acid was positively correlated with circulating levels of PYY. Nevertheless, this does not mean that PYY has no definite role in the appetite-suppressant effect of (other components of) whey proteins (e.g., GMP, glycomacropeptide), which, in the present study and others, stimulated a higher secretion of PYY than that ensuing the intake of maltodextrin [6,9].

Other interesting results were found in the present study. An example is represented by the triple correlation of citrulline with hunger (*r* = 0.537, *p* = 0.006), satiety (*r* = −0.480, *p* = 0.017) and GLP-1 (*r* = −0.530, *p* = 0.009), when considering obese subjects administered with maltodextrins. Due to high values of correlation and significance, this would suggest that citrulline plays a role in the stimulation of appetite and/or suppression of GLP-1 secretion after the ingestion of maltodextrins. Because of the great interest concerning the debated relationship between obesity and sugar-sweetened beverages in the scientific community [41], the biochemical and pharmacological properties of citrulline should be further investigated.

## 5. Conclusions

Eight specific amino acids (isoleucine, leucine, lysine, methionine, phenylalanine, proline, tyrosine, and valine) are implied in the appetite-suppressant and GLP-1-stimulating effects of whey proteins, which may be mediated by their binding with nutrient-sensing receptors expressed by L cells within the gastrointestinal wall. The long-term satiating effect of whey proteins and the effectiveness of a supplement with these amino acids (i.e., as a nutraceutical intervention) administered during BWRPs might deserve to be investigated in future studies.

## Figures and Tables

**Table 1:** Post-prandial responses in circulating levels of amino acids (expressed as AUC_T0−T120_ [×10^3^ µM × min]) after ingestion of a beverage containing whey proteins or maltodextrins in obese subjects (at T0).

Amino Acid	Maltodextrins	Whey Proteins	*p*
Alanine	41.0 ± 5.9	47.5 ± 6.0	0.015
Arginine	13.6 ± 2.1	15.9 ± 2.4	0.022
Asparagine	5.7 ± 1.1	8.9 ± 1.8	<0.001
Aspartate	3.2 ± 0.3	3.6 ± 0.6	0.096
Citrulline	1.9 ± 0.6	2.e9 ± 0.6	<0.001
Glutamate	15.5 ± 3.3	18.2 ± 6.7	0.144
Glutamine	42.7 ± 7.1	50.5 ± 8.1	<0.001
Glycine	22.3 ± 4.7	22.8 ± 5.9	0.623
Histidine	8.0 ± 0.9	8.9 ± 1.3	0.004
Hydroxyproline	0.6 ± 0.2	2.2 ± 1.5	0.022
Isoleucine	8.1 ± 2.0	20.2 ± 2.6	<0.001
Leucine	13.8 ± 3.5	33.5 ± 4.6	<0.001
Lysine	22.0 ± 3.2	36.6 ± 4.9	<0.001
Methionine	2.2 ± 0.4	4.0 ± 0.5	<0.001
Ornithine	6.3 ± 1.5	8.4 ± 1.4	<0.001
Phenylalanine	7.4 ± 1.3	9.6 ± 1.1	<0.001
Proline	18.7 ± 4.1	30.4 ± 5.0	<0.001
Serine	15.5 ± 2.1	19.1 ± 3.2	0.002
Taurine	9.0 ± 1.6	9.5 ± 2.0	0.556
Threonine	14.0 ± 3.8	22.0 ± 4.6	<0.001
Tyrosine	6.5 ± 1.4	10.8 ± 2.0	<0.001
Valine	26.3 ± 3.4	42.9 ± 4.8	<0.001

**Table 2:** Correlations of circulating levels of amino acids with hunger, satiety, PYY, and GLP-1 before and after ingestion of a beverage containing maltodextrins or whey proteins in obese subjects (all data: T0, T60, and T120).

Amino Acid	Maltodextrins				Whey Proteins			
	Hunger	Satiety	PYY	GLP-1	Hunger	Satiety	PYY	GLP-1
Alanine	0.368	−0.161	−0.087	0.080	−0.185	0.191	−0.343	0.408
	0.076	0.454	0.692	0.714	0.388	0.371	0.109	0.053
Arginine	−0.003	−0.032	−0.512	0.398	−0.329	0.412	−0.158	0.360
	0.987	0.880	0.012	0.060	0.116	0.045	0.471	0.091
Asparagine	0.001	0.019	−0.223	0.319	−0.416	0.372	−0.040	0.502
	0.993	0.929	0.307	0.138	0.043	0.073	0.856	0.014
Aspartate	0.171	−0.359	−0.292	0.044	−0.186	0.248	0.323	−0.179
	0.426	0.084	0.177	0.841	0.385	0.243	0.133	0.414
Citrulline	0.537	−0.480	−0.191	−0.530	−0.241	0.245	−0.036	−0.345
	0.006	0.017	0.382	0.009	0.257	0.249	0.867	0.107
Glutamate	0.540	−0.190	0.010	0.023	−0.108	0.206	0.453	−0.064
	0.006	0.375	0.963	0.915	0.614	0.334	0.029	0.770
Glutamine	0.091	−0.060	−0.202	0.188	−0.007	0.020	−0.103	0.360
	0.671	0.778	0.355	0.391	0.718	0.926	0.638	0.091
Glycine	0.049	−0.137	−0.169	−0.104	−0.020	−0.018	−0.312	−0.336
	0.819	0.524	0.441	0.638	0.923	0.933	0.148	0.117
Histidine	0.081	−0.052	−0.270	−0.085	−0.021	0.173	0.076	0.406
	0.704	0.807	0.212	0.697	0.919	0.419	0.729	0.054
Hydroxyproline	0.360	−0.204	−0.482	−0.313	−0.022	0.024	−0.168	0.627
	0.084	0.339	0.019	0.146	0.919	0.909	0.443	0.001
Isoleucine	−0.103	−0.221	−0.044	0.287	−0.630	0.572	0.311	0.724
	0.632	0.299	0.839	0.184	<0.001	0.003	0.149	<0.001
Leucine	−0.108	−0.216	0.014	0.248	−0.573	0.536	0.275	0.704
	0.614	0.311	0.949	0.254	0.003	0.006	0.205	<0.001
Lysine	−0.067	0.037	−0.185	0.656	−0.623	0.581	0.138	0.758
	0.755	0.862	0.399	<0.001	0.001	0.002	0.530	<0.001
Methionine	0.069	−0.041	−0.259	0.644	−0.569	0.472	0.074	0.656
	0.748	0.848	0.233	<0.001	0.003	0.019	0.737	<0.001
Ornithine	0.233	−0.119	−0.057	0.397	−0.223	0.134	0.018	0.184
	0.274	0.579	0.793	0.060	0.296	0.533	0.934	0.400
Phenylalanine	−0.054	−0.213	0.116	0.424	−0.503	0.436	0.246	0.548
	0.799	0.318	0.597	0.043	0.012	0.033	0.258	0.006
Proline	0.026	0.054	−0.021	0.697	−0.461	0.452	0.120	0.837
	0.901	0.802	0.923	<0.001	0.023	0.026	0.586	<0.001
Serine	−0.097	−0.057	−0.441	0.232	−0.262	0.194	−0.125	0.223
	0.650	0.791	0.035	0.286	0.216	0.365	0.570	0.306
Taurine	0.122	−0.295	0.099	0.186	−0.008	−0.085	−0.377	0.156
	0.569	0.162	0.651	0.396	0.969	0.692	0.076	0.478
Threonine	0.139	0.095	−0.681	−0.029	−0.292	0.365	−0.009	0.523
	0.518	0.658	<0.001	0.894	0.167	0.079	0.966	0.010
Tyrosine	0.539	−0.234	0.047	0.287	−0.561	0.568	0.260	0.423
	0.006	0.272	0.831	0.185	0.004	0.003	0.231	0.044
Valine	0.041	−0.346	0.072	0.387	−0.623	0.554	0.251	0.713
	0.848	0.097	0.742	0.068	0.001	0.005	0.248	<0.001

Note: first row represents r value, while second row *p* value.

## Abbreviations

BCAA	branched-chain amino acid
BMI	body mass index
CV	coefficient of variation
DPP-IV	dipeptidyl protease IV
FFM	fat-free mass
FM	fat mass
GLP-1	glucagon-like peptide 1
GMP	glycomacropeptide
PYY	peptide YY
SD	standard deviation
TAA	total amino acids
VAS	visual analogue scale
